# Supporting the self-management of hypertension: Patients' experiences of using a mobile phone-based system

**DOI:** 10.1038/jhh.2015.37

**Published:** 2015-04-23

**Authors:** I Hallberg, A Ranerup, K Kjellgren

**Affiliations:** 1 Institute of Health and Care Sciences, Sahlgrenska Academy, University of Gothenburg, Gothenburg, Sweden; 2 Centre for Person-Centred Care (GPCC), Sahlgrenska Academy, University of Gothenburg, Gothenburg, Sweden; 3 Department of Applied IT, University of Gothenburg, Gothenburg, Sweden

## Abstract

Globally, hypertension is poorly controlled and its treatment consists mainly of preventive behavior, adherence to treatment and risk-factor management. The aim of this study was to explore patients' experiences of an interactive mobile phone-based system designed to support the self-management of hypertension. Forty-nine patients were interviewed about their experiences of using the self-management system for 8 weeks regarding: (i) daily answers on self-report questions concerning lifestyle, well-being, symptoms, medication intake and side effects; (ii) results of home blood-pressure measurements; (iii) reminders and motivational messages; and (iv) access to a web-based platform for visualization of the self-reports. The audio-recorded interviews were analyzed using qualitative thematic analysis. The patients considered the self-management system relevant for the follow-up of hypertension and found it easy to use, but some provided insight into issues for improvement. They felt that using the system offered benefits, for example, increasing their participation during follow-up consultations; they further perceived that it helped them gain understanding of the interplay between blood pressure and daily life, which resulted in increased motivation to follow treatment. Increased awareness of the importance of adhering to prescribed treatment may be a way to minimize the cardiovascular risks of hypertension.

## Introduction

Extensive research has shown that hypertension is poorly controlled, and that treatment is a question of preventive behavior and risk-factor management.^1^ Efforts are needed to develop methods to support patients in self-managing their treatment.^2, 3^ It is a commonly held opinion that hypertension is a symptomless condition. However, the prevalence of headache and dizziness may be as high as about 57%, and correlates with blood pressure in both untreated and treated patients.^4^ Poor understanding of the relation between blood pressure, symptoms and lifestyle may contribute to poor outcomes of antihypertensive treatment.^5^

Mobile health (mHealth) technology^6^ offers new opportunities, and can be a way to support self-management behavior.^7^ Actively engaging patients in their care may improve outcomes, save money by avoiding unnecessary treatment, and reduce the number of office visits.^6^ mHealth may also enhance communication between health-care professionals and the patient, and thereby support self-management.^8^ A number of studies have reported improved blood-pressure control using mHealth solutions. For example, a mobile phone-based automated medication reminder application was shown to significantly improve adherence and blood-pressure levels among high-risk patients with hypertension.^9^ Moreover, Logan *et al.*^10, 11^ reported improved blood-pressure control with a mobile phone-based blood-pressure monitoring system. On the other hand, recent Cochrane reviews^12, 13^ conclude that there is limited evidence that self-monitoring and mobile phone messaging interventions provide benefit in supporting long-term illnesses. The best effect on blood-pressure control was found for treatment regimens in which self-monitoring was combined with education and/or counselling.^13^ The reviews conclude that, although research results to date are promising, there is a need for further research into these issues.

This study is part of a research program aiming to develop and evaluate an interactive mobile phone-based system aimed at supporting self-management in hypertension.^14^ The overall aim of this paper was to explore patients' experiences of using this self-management system.

Specific research questions were:

1. What were the patients' experiences of the technology used in the self-management system?
2. Did the patients feel that the system helped them self-manage their hypertension; and if so, how?

## Patients and methods

### Person-centered perspective

An important perspective in the research program is person-centeredness; that is, emphasizing the value of the patient's own experiences of his/her situation and personal resources.^15^ The patient is seen as a dignified and capable person, and it is of value to find a common ground and shared responsibilities between the patient and professionals.^16^ Three of the core concepts of person-centered care have been used: (i) *partnership and participation*, taking into account patient views; (ii) experiences of high blood pressure through *patient reports*; and (iii) *documentation* of patient reports in the mobile phone.^15^ The interdisciplinary group of researchers has also been guided by the US Food and Drug Administration's framework for the development of patient-reported outcome measures,^17^ by taking into account professionals' and patients' views about what is important to be aware of in daily life with hypertension.^18^

### Patients

Interviews were conducted with 49 of the 51 patients who had actively used the system for an 8-week period. The inclusion criteria for the patients were: >30 years, currently taking medication for hypertension, and having a mobile phone with an Internet connection. The patients were referred to the study by their treating health-care professional from four different primary health-care centers in southern Sweden. In total, 51 patients were included in the study. The study sample was comparable with a general hypertensive population in Sweden^19^ with respect to age and gender.

### Intervention

**Figure 1:**
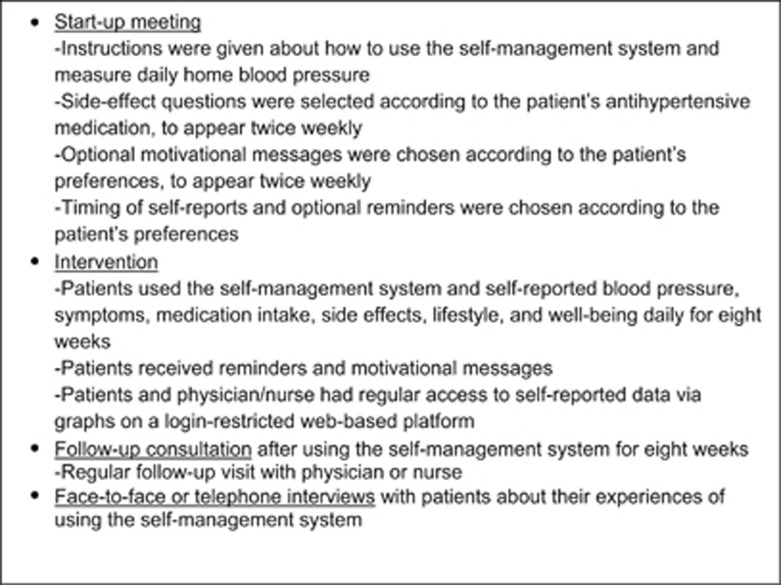
Overview of the study procedures.

Detailed descriptions of the development of the self-management system can be found elsewhere.^14, 20^ The self-management system has three main components: (i) a mobile phone platform for self-reporting blood pressure, symptoms, medication use, medication side effects, lifestyle behaviors and well-being, and for receiving tailored reminders and motivational messages; (ii) a device for measuring blood pressure; and (iii) a web-based feedback system showing graphs of selected self-reported data. The patients' self-reports were made once a day for 8 weeks. The study procedures and the intervention are briefly summarized in Figure 1. The self-management system has been shown to be reliable, and to efficiently and effectively capture information relevant for patients with hypertension.^21^

A communication system for mobile phones, Circadian Questions, was used. The system, developed by 21st Century Mobile, Stockholm, Sweden (http://www.cqmobil.se), supports platforms including Java J2ME, iPhone and Androids, and from 2013, also newer editions of Windows Mobile. In this study, the patient's own mobile phone was used. The technology is based on data traffic, which is more cost-effective than SMS and also a secure way to handle data.

Considerable emphasis was placed on the standardization of procedures outlined in the study protocol.^14^ Patients were trained in how to measure their blood pressure, according to the European Society of Hypertension Practice guidelines for home blood-pressure monitoring.^22^ A home blood-pressure monitor (BP A200 AFIB; Microlife USA Inc., Clearwater, FL, USA), validated according to the international protocol of the European Society of Hypertension, was used.^23^

### Data collection

Semi-structured and audio-recorded face-to-face interviews (*n*=25) or telephone interviews (*n*=24) with the patients were conducted after completion of the intervention. The interviews lasted an average of 29 min (range 13–63 min). Examples of interview questions are: ‘What are your experiences from participating in the mobile phone project?', ‘How did it work to measure your blood pressure and report in your mobile phone?' ‘How did it work to look at the graphs?' and ‘Were the motivational messages helpful?' Interviewers posed probing questions to deepen, clarify and develop the responses. Data were collected from April to June 2012.

### Data analysis

The interviews were transcribed verbatim and coded using NVivo 10 (QSR International Pty Ltd, Doncaster, VIC, Australia), a qualitative research software program designed to help users organize and analyze nonnumerical data. Qualitative thematic analysis was used to analyze data.^24^ Thematic analysis, which allows working with both a deductive and an inductive approach, was chosen as an appropriate method for identifying, analyzing, interpreting and reporting themes. The analyses focused on themes addressed in the interviews regarding patients' experiences from the technology and how they felt the system had helped them self-manage their hypertension.

We developed a coding framework to organize the data systematically based on analyses of the transcripts. When searching for themes, we considered how they could be combined into sub-themes from which overarching themes could be derived. We reviewed the suggested themes to determine whether they worked in relation to our codes and whether they were valid in relation to the entire data set, by reading all assembled quotes for each theme. Codes were organized into sub-themes, from which overarching themes were derived. To present and visualize the results, quotes were identified.^24^ The analysis was iterative, and was performed by the first author in collaboration with the co-authors and discussed to reach agreement in our understanding of the data.

### Ethical considerations

The project, approved by the Regional Ethics Board in Gothenburg, Sweden (study codes 551-09 and T-100-12), was planned and conducted in accordance with the Declaration of Helsinki.^25^ All patients were informed about the study both in writing and orally before giving their written informed consent. Transcripts were anonymized, and the patients were ensured confidentiality. The study was registered in the Clinical Trial Protocol Registration System (ClinicalTrials.gov NCT01510301), under the acronym MIHM (Mobile phone In Hypertension Management), and was further monitored by an independent monitoring board.

## Results

Forty-nine of the 51 included patients completed the study with an interview after 8 weeks. One patient dropped out 4 weeks into the study, and one was not available for interview after the study. Patient characteristics are presented in Table 1.

**Figure 2:**
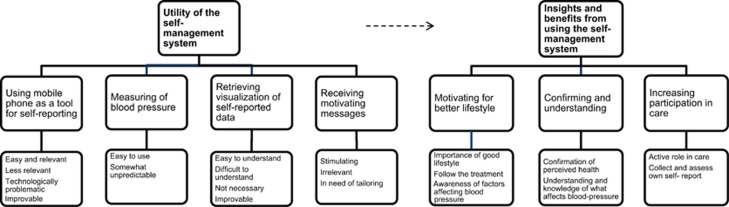
Overview of the sub-themes and overarching themes. The figure shows the relation between the themes.

The analysis resulted in two themes: *Utility of the self-management system* and *Insights and benefits from using the self-management system*. The sub-themes within each theme are displayed in Figure 2.

### Utility of the self-management system

This theme focuses on the patients' experiences of the technology used.

#### Using mobile phone as a tool for self-reporting

Almost all the patients felt it was easy to self-report in the mobile phone, and that the questions were relevant. They also pointed out that it was much easier to use a mobile phone than log into a computer to answer questions:

‘It's been great, easy, outstanding. Easier than logging on to my computer.' (Male Patient (Pt) number 32)

However, patients who did not perceive any symptoms or who had stable blood pressure found some questions to be less relevant. Technical difficulties in the startup of their participation were reported. A few others mentioned a weakness in the form of connection problems when using their mobile phone. Problems with self-reporting were mentioned, such as how to go back and change an answer that had been registered. More outright suggestions for improvement included offering a better manual. Other patients wanted the option to add comments to their answers (for example, ‘I have the flu today, so I feel tired and have a headache') and more reminders of how to answer in case they had forgotten how to do this.

#### Measuring of blood pressure

For many of the patients, using the blood-pressure monitor was also easy in conjunction with reporting using their mobile phones. Nevertheless, a few perceived a somewhat unpredictable weakness in the form of problems using the monitor, which sometimes by accident measured their blood pressure more than three times if they mistakenly touched the start button:

‘Sometimes the monitor measured more than three times, but I might not be sitting still, but otherwise there were no problems.' (Male Pt 11)

#### Retrieving visualization of self-reported data

Many different views were expressed about the graphs; for example, some felt it was easy to read, interpret and understand them, whereas others found it difficult. Another weakness was that, for some, simply logging into the computer to look at the graphs was a problem in itself because they had forgotten how to do this. Therefore, some patients did not in fact look at the graphs themselves in their own homes but instead did so during their visit to the clinic:

‘Haven't looked at the graphs beforehand because I'm not good with computers, but I've kept it in mind and thought about what I've done on the days when my blood pressure has been high…when I looked [at the graphs] with the nurse it wasn't hard to see.' (Female Pt 47)

However, some said they did not consider it necessary to look at the graphs, as their answers and values were quite similar without significant variation. Others suggested improvement in how the graphs were displayed; for example, using different colors for the variables. They also needed some help interpreting the results. A suggestion was made for improvement in the form of a question about whether one wants to look at the graphs in the mobile phone directly after answering the questions.

#### Receiving motivational messages

Patients expressed different views about receiving motivational messages in their mobile phones. Many described them as stimulating and creating a feeling of getting ‘pepped up', whereas others ignored them or had chosen not to receive them at the beginning of their participation in the research project:

‘It was good…for example ‘Made food today?' Even if I didn't need them there was a thought…that made you think about it and stuff…I think it's pretty good to have those kinds of questions, kind of encouraging.' (Female Pt 23)

Suggestions for improvement included having the option to formulate one's own motivational messages instead of choosing from a repertoire provided in the mobile phone platform. Another idea involved tailoring messages to particular answers, such as when a patient reported less exercise than advisable, he/she would receive motivational messages about increasing it; or, alternatively, when one had exercised sufficiently, receiving a message when this had in fact influenced one's blood pressure positively.

### Insights and benefits from using the self-management system

This theme describes how the patients perceived that the system had helped them self-manage their hypertension.

#### Motivating for a better lifestyle

Patients reported greater insight and motivation to manage their health through lifestyle changes and adherence to treatment when using the self-management system. Almost all the patients had experienced having an ‘eye-opener' regarding the importance of good lifestyle with regard to their hypertension. Others said they had made lifestyle changes, such as losing weight or quitting smoking. Follow-up was perceived as important for feeling motivated to follow the treatment. One of the patients expressed this as follows:

‘Follow-up is incredibly important for feeling motivated to follow the treatment. Sometimes it can be pretty annoying to take medicine and you can think ‘Nah, I felt pretty good before' and just not do it.' (Female Pt 12)

Many of the patients also received confirmation that they needed to take their medicine to keep their blood pressure at a normal level. This became evident to those who missed their antihypertensive medication at some point and then personally detected that their blood pressure had gone up the same day:

‘Yeah, I saw an effect [in the graphs] when I forgot my medication one day; that taught me a lesson, I never thought it could be seen only by forgetting one day. I was surprised; it's almost a little frightening. The consequence will be that I'll be less sloppy in the future.' (Male Pt 13)

Patients were motivated to follow their treatment when they became aware of the various factors affecting blood pressure, either via the graphs or through answering questions and measuring their blood pressure. In addition, some patients used their own notebook to make notes of their measured blood-pressure values and how they felt.

#### Confirming and understanding

Many patients felt they had received confirmation of the interplay between blood pressure, symptoms and perceived health, and of how changes in blood pressure depended on one's state of health and what happened in everyday life:

‘I've gained more insight into things that affect my well-being. That's very valuable indeed. It's not easy to pinpoint how one feels or the reasons why - so the graphs really helped to show how things were related to one another [...] They helped me understand why things turn out the way they do, what affects me, and so now I feel I have better control, which I haven't felt before.' (Male Pt 31)

The interplay between physical activity, stress and blood pressure was the most obvious insight for the patients. Although they knew in a general sense that physical activity is good for decreasing high blood pressure, this became more obvious and they gained new knowledge that it really is true:

‘The most important thing I've learned is that you should be more physically active; I've never really thought about or known that…If you're more physically active then…yeah, maybe I've read about it in health magazines…but haven't reflected that much on it like this…now you can actually see it on the curve…you get motivated to do something beyond the norm.' (Male Pt 38)

Other patients did not experience the self-management system as very useful, mainly because they had had stable blood pressure or had not perceived any symptoms. Still others believed it could be very useful and valuable when new antihypertensive medicine was prescribed, when their dose was adjusted, during periods of perceived symptoms, or as an aid in getting motivated for treatment.

#### Increasing participation in care

The patients mentioned feeling more involved in the follow-up consultation after using the self-management system. They described their participation during the visit as playing a more active role in the conversation and taking more responsibility for discussing their health, compared with previous health-care visits. Moreover, they perceived it as a better and more meaningful consultation as the graphs functioned as a common ground for discussion. Also, patients said they could now take more responsibility for their own care:

‘I mean I've lived with this high blood pressure a long time and sometimes I can have quite bad headaches, and I don't think much about it, I'm so used to it; but here I really think and consider how I feel, and realize I actually have to try to do something about it.' (Female Pt 17)

The self-reporting was generally perceived as a source of support and as valuable for discussing how one's well-being, symptoms and lifestyle had varied over the study period, even if the patient not had looked at the graphs at home. Overall, the patients perceived it as positive to self-report and assess how their blood pressure, symptoms and lifestyle varied from day to day:

‘Normally you go for a visit […] and they check your blood pressure and just say it's good, but I don't know what would be good or bad, really. Now I know more; that gives me an awareness of how my body works. Yes, now the visit's different for both me and the nurse. Now I had information collected over a longer period of time; before it's only been about when you're there [at the visit]…' (Male Pt 42)

## Discussion

Using the self-management system gave insights and perceived benefits that led to an increased awareness of the factors that influence blood pressure. The interplay between blood pressure, stress and physical activity was the most obvious insight. The possibility to see how one's blood pressure is affected by one's daily life situation, lifestyle and medication may motivate adherence. This is in concordance with a study by Bokhour *et al.*,^26^ who report that hypertensive patients' ability to perform self-management is affected by their daily life experiences. From a general practitioner's perspective, Howes *et al.*^27^ recently identified barriers to lifestyle management in hypertension. Patient reluctance and ambivalence to follow treatment advice were identified as major barriers affecting patients' ability to initiate and maintain lifestyle changes. They conclude that many interventions to decrease blood pressure in the past may have failed owing to a lack of understanding of the underlying barriers, for example, motivation to make lifestyle changes. The challenge now is therefore how to best address these barriers.

The importance of effective communication has previously been emphasized as a prerequisite for a successful encounter between patient and health-care professional.^8, 28^ According to the patients' experiences, the self-management system we have developed offered possibilities for a more active and responsible role in the follow-up consultation. Further, the patients perceived that the self-management system contributed to a better and a more meaningful discussion at the consultation thanks to the graphs, which visualized factors affecting their blood pressure. We argue that the use of mHealth via a mobile phone platform would further strengthen patient involvement in care. mHealth offers new opportunities to manage healthcare, but little is known about the impact of interventions on outcomes.^12, 13^ The possibilities for better management using new technology and the importance of increased patient participation have been pointed out in several new guidelines for the treatment of hypertension.^29, 30^

In general, the patients perceived the self-management system as easy and relevant to use. This kind of platform for mobile phone-based self-management must function as a whole in a practical situation with all its parts. The participating patients' attitude toward using new technology for self-monitoring a treatment regimen is important. The design shall reflect the needs of the users,^31^ our results show a technical solution and a situation in which this seems to have been the case. The results also provide insight into potential issues for improvement. Worth noting is that for some patients, reporting in the mobile phone and connecting to it as well as to the computer to look at the graphs can still be somewhat difficult. For example, the self-management system could be more useful if the graphs were adjusted for viewing on the mobile phone. Other ideas for improvement involved, for example, bettering the manual as well as offering the opportunity to tailor questions and graphs according to personal preferences to increase the usefulness for the individual patient. In line with this, reminders and motivating messages should be tuned according to answers. This is especially important, we argue, to ensure that all groups of patients will use the system to reach positive health effects. This potential improvement is therefore important, but would demand a somewhat more advanced technological solution with the capacity to continuously react to patient reports of data.

In sum, patients' experiences and insights gained from using the self-management system are thus multifaceted and entail various aspects (Figure 2). It is important to remember that the insights gained and the activities enabled for patients in the form of increased adherence, changed lifestyle and increased participation in care (for example, during follow-up consultations) depend on the actual *use* of some or all of the components of the self-management system.

Person-centered care is an increasingly important perspective in today's healthcare.^32^ The importance of engaging patients as active and responsible partners in their own care and in the decision-making about it have been shown to be of high value. The results of our project show a concrete way to situate person-centered care by taking into account patients' views and experiences. An even more important way is the layout of the self-management system, which puts patient reports and documentation^15^ in the hands of both professionals and patients. In this manner, our results provide an example of how this person-centered approach can be applied in practice, and give some indication of the effects of care. More specifically, McManus *et al.*^33^ recently showed that allowing hypertensive patients at high risk of cardiovascular disease to be more active in their care through self-monitoring and medication self-titration resulted in lower systolic blood pressure.

Socioeconomic issues should be addressed further. Stoddart *et al.*^34^ reported that the telemonitoring-based management of uncontrolled hypertension was both significantly more expensive and more effective than usual care, and recommended a long-term modeling of costs to examine the cost-effectiveness implications. The use of the patient's own mobile phone and data traffic for self-reports probably makes our system cost-effective. However, economic evaluation is important in future research.

The experience from the different interview methods is that interviewing face-to-face allowed more in-depth insights through visual cues, for instance, how to proceed with the dialogue or whether to ask follow-up questions.^35^ The telephone interviews allowed the patients to be interviewed at home, in the same environment where they had used the self-management system. Further, interviewing by telephone made it possible to validate the results from the face-to-face interviews. Thus, we saw the different modalities as complementary and useful in this context.

The strength of this study is its use of a qualitative method to generate rich insight into patients' experiences from using the self-management system daily for 8 weeks. Thematic analysis was considered appropriate for analyzing the data, owing to the method's flexibility regarding inductive and deductive approaches.^24^ Furthermore, the method is well suited to large data sets. Credibility was addressed through critical judgment, with all authors taking an active part in the data analysis. The analyses were discussed at several meetings, until consensus was reached. Transferability was addressed through describing the context and characteristics of the patients. The use of quotations to illustrate the findings may help the reader judge the relevance of the interpretation.^36^

The study's limitations include the selection bias of the patients. Like in many other studies, it is often the responsive patients who are already adhering to treatment who are included. Non-adherent patients, for whom the intervention is potentially most beneficial, are often not willing to participate; had they participated, they would have most likely reported other experiences. The findings from this qualitative study cannot be generalized to all patients with hypertension, but may generate hypotheses for further research.

In conclusion, the study shows that the mobile phone-based system is useful for patients in self-managing their hypertension. It gave them insights, motivation and an understanding to help them adhere to their treatment. Thus, our study has important implications for further research and clinical practice regarding the renewal of hypertension treatment as well as technological development. Furthermore, the study gives an example of how important it is to strengthen the patient's possibility to play an active and responsible role in his/her own care to minimize the cardiovascular risks of hypertension.


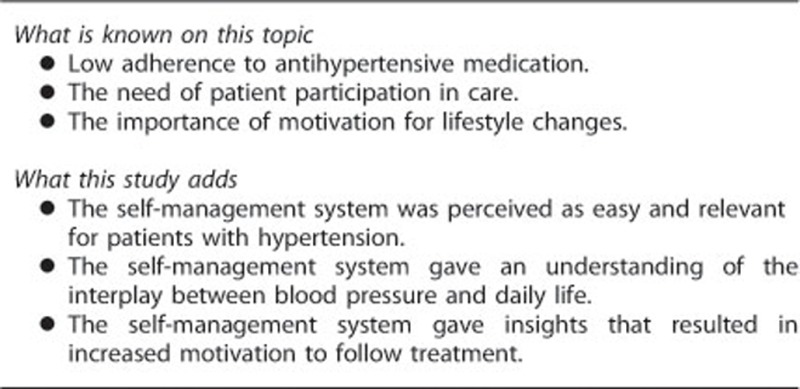


## Figures and Tables

**Table 1:** Patient characteristics

*Patients (*n*=49)*	*Females (*n*=23)*	*Males (*n*=26)*
Age, median (range)	58 (46–72)	62.5 (37–81)
Years with hypertension, median (range)	8 (<1–25)	6.5 (<1–32)
		
*Marital status*		
Married/cohabitating	16	22
Single	7	3
Widowed		1
		
*Education*		
Compulsory school (⩽ 9 years)	1	4
High school (9–12 years)	10	11
University	11	11
Missing	1	
		
*Employment status*		
Employed	13	14
Unemployed		1
Retired	8	11
Missing	2	
